# Nonalcoholic Fatty Liver Disease Relationship with Metabolic Syndrome in Class III Obesity Individuals

**DOI:** 10.1155/2015/839253

**Published:** 2015-05-18

**Authors:** A. Cordeiro, S. E. Pereira, C. J. Saboya, A. Ramalho

**Affiliations:** ^1^Micronutrients Research Center (NPqM), Faculty of Medicine, Federal University of Rio de Janeiro (UFRJ), 21941-590 Rio de Janeiro, RJ, Brazil; ^2^Micronutrients Research Center (NPqM), Faculty of Medicine, Federal University of Rio de Janeiro, 21941-590 Rio de Janeiro, RJ, Brazil; ^3^Federal University of São Paulo, 04021-001 São Paulo, SP, Brazil; ^4^Carlos Saboya Clinic, 22764-000 Rio de Janeiro, RJ, Brazil; ^5^FIOCRUZ, 21040-900 Rio de Janeiro, RJ, Brazil; ^6^Social Applied Nutrition Department, Micronutrients Research Center (NPqM), Federal University of Rio de Janeiro, Avenida Brigadeiro Trompowski, s/n 2° Andar, Bloco J, Ilha do Fundão, 21941-590 Rio de Janeiro, RJ, Brazil; ^7^Micronutrients Research Center (NPqM), Institute of Nutrition Josué de Castro (INJC), Federal University of Rio de Janeiro (UFRJ), Avenida Brigadeiro Trompowski, Subsolo, Bloco J, Ilha do Fundão, 21941-590 Rio de Janeiro, RJ, Brazil

## Abstract

*Introduction*. Obesity is represented mainly by abdominal obesity and insulin resistance (IR), both present in most individuals diagnosed with metabolic syndrome (MS). IR is the key risk factor in the pathogenesis of nonalcoholic fatty liver disease (NAFLD). *Objective*. To relate NAFLD to MS in class III obese individuals. *Methodology*. A descriptive cross-sectional study with class III obese individuals, aged ≥ 20–60 years. Blood pressure measurement, weight, height, body mass index (BMI), waist circumference (WC) and blood glucose, insulin, high-density lipoprotein cholesterol (HDL-c), and triglycerides data were obtained. HOMA-IR (homeostatic model assessment insulin resistance) calculation was carried out with a cutoff value of 2.71 for IR evaluation. The diagnosis of NAFLD was performed by liver biopsy and the diagnosis of MS was performed in accordance with the National Cholesterol Education Program/Adult Treatment Panel III (NCEPATP III). *Results*. Of the 50 individuals evaluated, 86% were women and BMI means were 45.4 ± 3.6 Kg/m^2^. The overall individuals had NAFLD, 70% steatosis, and 30% steatohepatitis. The diagnosis of MS occurred in 56% but showed no significant association with NAFLD (*P* = 0.254). Triglycerides (178 ± 65.5 mg/dL) and insulin (28.2 ± 22.6 mcU/mL) mean values were significantly higher in steatohepatitis (*P* = 0.002 and *P* = 0.042, resp.) compared to individuals with steatosis. IR was confirmed in 76% and showed a relationship with NAFLD severity. *Conclusion*. NAFLD was not related to MS; however, MS components, evaluated in isolation, as well as IR, were related to the presence and severity of NAFLD.

## 1. Introduction

The nonalcoholic fatty liver disease (NAFLD) is a term used to describe a clinical pathological condition characterized by deposition of triglycerides in hepatocytes, exceeding 5–10% of that organ weight [1]. It is estimated that NAFLD prevalence in the world ranges from 20 to 30% [2]. In Brazil, this index is still unknown, but the Brazilian Society of Hepatology [3] presented a study in which the frequency of steatosis, nonalcoholic steatohepatitis, and cirrhosis was, respectively, 48%, 37%, and 5%.

As obesity prevalence increases, NAFLD prevalence also increases, and there are projections that it will become the most common liver disease form. NAFLD occurs in 40–80% of the obesity cases with body fat distribution being more important in NAFLD than total body fat, since the accumulation of visceral fat has been reported to be more related to the development of hepatic steatosis [4]. Lira and coworkers [5] described a strong correlation between NAFLD presence and metabolic changes in obese patients. The odds ratio for NAFLD development in this group was 10.77 regarding eutrophic individuals. Even if it is not part of the diagnostic criteria for metabolic syndrome (MS), NAFLD is often associated with this metabolic disorder [6].

MS is a condition intensely related to the global epidemic of obesity, since in its physiopathological basis the excess of visceral fat is a precursor of metabolic changes. It can be defined as a group of interrelated risk factors of metabolic origin which directly contributes to the development of cardiovascular disease (CVD) and/or diabetes mellitus type 2 (DM2). Still it has not been established whether MS development is due to a single cause or to multiple causes, but it has been acknowledged that abdominal obesity and insulin resistance (IR) seem to play a fundamental role in this syndrome genesis [7].

Considering that NAFLD relationship with MS and with its constituent components has been increasingly acknowledged, such findings have stimulated the interest in studies focusing the role played by liver disease on the increase of cardiometabolic risk, aspects that constitute the objective of the present investigation.

## 2. Methods

Class III obesity individuals, of both sexes, aged ≥ 20 to 60 years, with indication for bariatric surgery, were included in this study. These patients were from a clinic specialized in controlling obesity in Rio de Janeiro municipality, being the study conducted from August 2012 to October 2013. Pregnant women, nursing mothers, and patients with malabsorption bowel syndrome, acute and chronic infections, alcohol consumption exceeding 20 g/day in women and 40 g/day in men, and any other liver disease, except NAFLD, were excluded from the study.

### 2.1. Anthropometric Evaluation

Class III obesity classification was based on the World Health Organization (WHO) criteria [8]. BMI calculation was conducted using the weight (kg) and height (m^2^) [9] anthropometric measurements.

Waist circumference (WC) measurement was performed with the patient in a standing position with abdomen relaxed, arms at the sides, and feet together, using a nonextensible tape measure. The tape involved the individual in the largest abdominal diameter. The measurement was carried out at the completion of the patient's normal expiration [10].

### 2.2. Biochemical Evaluation

For biochemical evaluation, blood sample was obtained by venipuncture, after a 12 h fast period. Laboratory tests were conducted to evaluate metabolism and liver function and damage: albumin, aspartate aminotransferase (AST), alanine aminotransferase (ALT), alkaline phosphatase (AP), gamma-glutamyl transpeptidase (GGT), lipid profile [total cholesterol, triglycerides, high-density lipoprotein cholesterol (HDL-c), low-density lipoprotein cholesterol (LDL-c)], glucose, and insulin. Determinations of triglycerides, total cholesterol, HDL-c, and glucose were carried out by enzymatic colorimetric method.

Reagents for these biochemical evaluations were purchased from Labtest Diagnóstica S.A., Minas Gerais, Brazil. The LDL-c fraction was determined in accordance with Friedewald's formula. Basal insulin was quantified by reversed-phase high performance liquid chromatography (RP-HPLC) (Labtest Diagnóstica S.A., Minas Gerais, Brazil). The cutoff points for total cholesterol and fractions, triglycerides, and glucose were those established by the NCEP-ATP III criteria for diagnosing MS, a disease confirmed when there is presence of three or more risk factors [11], as shown in Table 1.

IR was identified by the HOMA-IR index [12] obtained from the following calculation: HOMA-IR = fasting insulinemia (mU/L) × fasting blood glucose (mmol/L)/22.5. The reference values used were found in the literature for adult individuals [13], and the value above 2.71 was the cutoff point.

### 2.3. Systemic Blood Pressure Measurement

The blood pressure measurement by indirect method was conducted and the OMRON HEM 705 CP portable tensiometers (OMRON Healthcare Europe B.V., Hoofddorp, Netherlands) were used with appropriate clamps to measure the brachial circumference of the patient with a range of 0 to 300 mmHg and an accuracy of ±3 mmHg. The overall technical requirements for obtaining the appropriate systemic blood pressure, as well as the definition of the cutoff point equal to or above 130/85 mmHg that is already considered prehypertensive, obeyed the VI Brazilian Guidelines of Arterial Hypertension specifications (Diretrizes Brasileiras de Hipertensão Arterial VI-2010) [14].

### 2.4. The Diagnosis of NAFLD: Liver Biopsy

Histological evaluation was performed by liver biopsy through the withdrawal of 4 mm of the liver left lobe thickness, obtained via puncture with a 16 G × 15 cm Menghini needle (Euromed, Minas Gerais, Brazil). Biopsies were conducted by the medical surgeon along the bariatric surgery. All histological evaluations were performed by the same pathologist, who had no knowledge of the patients' clinical and biochemical data, through hematoxylin-eosin, Masson and Perl's trichrome stainings (Interlab, São Paulo, Brazil). Hematoxylin-eosin allows a general view of acinar architecture, inflammatory infiltrates, and alterations in hepatocytes. Masson verifies the presence of fibrosis, be it portal, perisinusoidal, or around centrilobular veins and Perl verifies the presence of iron deposits [15].

NAFLD gradation and the staging of liver fibrosis were determined in accordance with the proposal of Brunt and coworkers [15]. Gradation was performed considering the presence of macrovesicular steatosis (simple steatosis), necroinflammatory activity (presence of steatohepatitis), and presence of fibrosis and cirrhosis.

### 2.5. Statistical Analysis

Statistical calculations were performed by the SPSS program version 17.0. The statistical analyses used were Student's *t*-test (for mean of unpaired samples), Pearson's Correlation, and Spearman's Correlation (between nonparametric variables), and association was verified by the chi-square (*χ* ^2^) test. A 5% significance level was considered.

This study was approved by the Research Ethics Committee of Hospital Universitário Clementino Fraga Filho of Universidade Federal do Rio de Janeiro (Research Protocol n° 011/06-CEP).

## 3. Results

The sample comprised 50 individuals, being 43 (86%) female and 7 (14%) male. Mean age was 43 ± 10.5 years, ranging from 26 to 60 years.  Table 2 shows the sample general characteristics.

Regarding WC, in accordance with the cutoff set by WHO [8], 100% of the patients had a very high risk of metabolic complications associated with obesity.

The diagnosis of NAFLD was confirmed in 100% of the patients according to histological evaluation data after liver biopsy: 70% of the individuals had steatosis and 30% had steatohepatitis. Among those who had steatohepatitis, 13% presented fibrosis.

Means of the anthropometric variables were observed according to the staging of NAFLD (Table 3).

Table 4 shows the mean serum concentrations of biochemical indicators of liver function and damage according to the staging of NAFLD. GGT activity was higher in individuals with steatohepatitis (significantly for steatohepatitis with fibrosis* versus* steatosis); ALT and AST liver enzyme activities were within the normality standard as well as AP levels. Albumin dosages were significantly lower in individuals with steatohepatitis with fibrosis.

Among the obese patients studied, 56% had MS and 72% had systemic arterial hypertension (SAH), but there was no significant association between MS and NAFLD (*P* = 0.254) or between NAFLD and SAH (*P* = 0.105).

Table 5 shows the mean serum concentrations of MS and IR biochemical indicators according to the staging of NAFLD, and it reveals that the highest insulin and triglycerides means were observed in steatohepatitis (significantly for steatohepatitis with fibrosis* versus* steatosis).

Among the obese individuals with steatohepatitis those who had fibrosis showed HDL-c mean below the recommended cutoff point for MS corresponding to 38.9 ± 7.2 mg/dL (*P* = 0.038).

It was found that 76% of the individuals had IR according to the HOMA-IR calculation, 68% of these individuals had steatosis, 32% had steatohepatitis, and individuals who had fibrosis also had IR.

## 4. Discussion

In this study, we observed that individuals with the highest mean of WC showed a significantly more advanced degree of NAFLD confirming previous results, where excess of fatness, particularly in the abdominal region (central obesity), is strongly associated with NAFLD and IR. This predisposes to SAH, dyslipidemia, and inflammation, factors present in MS. The association between these clinical evidences and the excess of adipose tissue involves metabolic and inflammatory mechanisms [16]. Intra-abdominal fat in eutrophic individuals constitutes around 10% of body deposits. It is formed by fat surrounding the internal viscera, the omentum, and mesentery [17], and the omentum and mesentery venous drainage is given by the portal venous system. Thus, the liver is directly exposed to cytokines and free fatty acids (FFAs) released by the visceral adipose tissue, while the same metabolites released by the adipose tissue from other regions reach the liver diluted by the systemic circulation. In the NAFLD “portal theory,” there is an exacerbated release of FFAs, endotoxins, and proinflammatory cytokines of visceral fat that reach the liver, via portal system, promoting the development of liver resistance to insulin, hepatic steatosis, and inflammation in obese individuals [16, 18].

In general, albumin is a good marker of liver disease severity and classically evaluates liver function [19]. In our study, serum albumin levels were lower in the group with steatohepatitis without fibrosis* versus* the steatosis group but still within the normality range. When individuals with steatohepatitis with fibrosis are considered, the albumin values found were even lower and just below the normality value recommended (3.4 g/dL). Such results are close to the values found by Sarma and coworkers [20] in patients with cirrhosis resulting from alcoholic etiology or cryptogenic origin (3.74 g/dL). Hypoalbuminemia is an important feature of liver disease worsening. Yennu and coworkers [21] observed that an albumin level less than 3.5 g/dL was an independent predictor of moderate to severe inflammation, as well as advanced fibrosis.

Among the hepatocellular damage markers mostly used in the literature are the transaminases, GGT, and AP [22]. In this study, the ALT, AST, and AP mean levels among individuals with hepatic steatosis and steatohepatitis showed no significant differences. Although many studies reported an increase in the liver function and damage markers in individuals with various degrees of NAFLD [23, 24], generally these enzymes are normal in over 78% of the individuals [25]. Furthermore, the increases described, most often, are moderate and are restricted to AST and ALT, suggesting that the diagnosis and severity of the disease cannot be defined solely by the evidence of liver damage. Papadia and coworkers [26] pointed out that the increase in AST and ALT means was trusted as a marker of the degree of fat infiltration in hepatocytes, with significant increase in individuals with triglycerides accumulation in the liver reaching over 70%, but not as a marker of inflammation and fibrosis. Lomonaco and coworkers [27] observed normal ALT levels in the overall NAFLD stages.

With regard to GGT values, the present study showed higher values in the individuals with steatohepatitis, significantly in the presence of fibrosis. These results are in line with the findings that demonstrate that liver disease worsening may be a possible explanation for GGT increase with the progression of liver fibrosis [28]. However, Silva and Duarte [29] found no association between GGT levels and hepatic steatosis, suggesting that the increase is due to GGT increased hepatic synthesis, like an adaptive answer to changes arising from the disease, associated with a greater GGT release into blood circulation.

Considering the MS biochemical components, hypertriglyceridemia and HDL-c low concentrations are the lipid profile impairments usually associated with the presence of NAFLD [30]. In the present study, HDL-c serum concentrations, as assessed in stages of liver disease, showed no significant changes. However, in individuals with steatohepatitis who also had fibrosis, HDL-c mean concentrations were significantly lower. Boza and coworkers [31] have observed significantly HDL-c lower means in class III obese individuals with NAFLD in comparison to the group without the disease, and this variable was the only lipid fraction associated with the diagnosis of NAFLD. Similarly, a study developed by Chaves and co-workers [24] reported that the only lipid fraction related to the presence of steatosis was HDL-c, showing significantly lower median in patients with NAFLD. In the study of Dias and coworkers [32], which assessed possible predictors of NAFLD in obese individuals, no correlation for lipid fractions was observed occurring in the most advanced stages of the liver disease. However, a weak negative correlation was observed between HDL-c levels and the degree of simple steatosis, classified as mild, moderate, or severe.

In the present study, we observed that triglycerides mean was higher in steatohepatitis (especially in fibrosis patients) as found in the study of Júnior and Nonino-Borges [33], which suggests that such changes are steatohepatitis predictors since triglycerides synthesis increases in patients with NAFLD, mainly in those with obesity and DM2. Triglyceride accumulation in the liver tissue occurs as a consequence of adipose tissue lipolysis and hepatic* de novo* lipogenesis [34]. Triglycerides can be exported from the liver by very low-density lipoprotein (VLDL) particles formed by the incorporation of triglycerides to apolipoprotein B (apoB) [35]; thus, changes in the synthesis and secretion of apoB also contribute to hepatic triglyceride accumulation [36].

Insulin mean was high in obese individuals with steatohepatitis (especially in fibrosis patients), and IR was higher (although not significant) in the most advanced stage of liver disease. This metabolic change is considered a key risk factor in NAFLD pathogenesis being connected with the increase in oxidative stress and lipotoxicity [37–39]. It is believed that IR, oxidative stress, and inflammatory cascade play an essential role in the disease pathogenesis and progression. In addition to these facts, there are a number of interactions between hepatocytes, stellate cells, fat cells, Kupffer cells, inflammatory mediators, and reactive oxygen species resulting in inflammation or cirrhosis. In IR states, fat and muscle cells have a preference for oxidizing lipids and a high proportion of the higher levels of FFA released from fat cells are taken by the liver, leading to steatosis [40]. Macrovesicular steatosis arises from the increased hepatic synthesis of fatty acids, esterification of these fatty acids in triglycerides, and decreased triglyceride transport out of the liver [41]. Peripheral resistance to insulin also contributes to the increased entry of FFAs in the liver, which causes unbalance between oxidation and export of FFAs, resulting in fat accumulation in the liver parenchyma [42].

## 5. Conclusion

NAFLD was not related to the diagnosis of MS; however, MS components, evaluated in isolation, as well as IR, which is often associated with MS, were related to the presence and severity of liver disease. These results suggest the importance of monitoring these components in the screening for NAFLD.

## Figures and Tables

**Table 1:** NCEP/ATP III criteria.

Components	Presence of ≥3 components
Glucose	≥100 mg/dL
HDL-c	<40 mg/dL for men <50 mg/dL for women
Triglycerides	≥150 mg/dL
WC	≥102 cm for men or ≥88 cm for women
Systemic arterial hypertension	≥130 × 85 mmHg

HDL-c: high-density lipoprotein cholesterol; WC: waist circumference.

**Table 2:** Sample general characteristics (*n* = 50).

Characteristics	Mean/SD
Age (years)	43.0 ± 10.5
BMI (Kg/m^2^)	44.1 ± 3.8
Weight (Kg)	121.4 ± 21.4
WC (cm)	121.6 ± 12.7
Albumin (g/dL)	4.2 ± 0.5
AST (U/L)	25.1 ± 18.3
ALT (U/L)	27.4 ± 15.9
AP (U/L)	76.1 ± 23.4
GGT (U/L)	33.4 ± 21.8
TG (mg/dL)	132.0 ± 50.9
C (mg/dL)	197.7 ± 36.1
LDL-c (mg/dL)	121.8 ± 34.5
HDL-c (mg/dL)	48.3 ± 11.9
Insulin (mcU/mL)	19.0 ± 12.5
Glucose (mg/dL)	100.2 ± 19.0
HOMA-IR	4.5 ± 4.7
SBP (mmHg)	180 × 125

AST: aspartate aminotransferase; ALT: alanine aminotransferase; AP: alkaline phosphatase; BMI: body mass index; C: total cholesterol; GGT: gamma-glutamyl transpeptidase; HDL-c: high-density lipoprotein cholesterol; HOMA-IR: homeostatic model assessment insulin resistance; LDL-c: low-density lipoprotein cholesterol; SBP: systemic blood pressure; TG: triglycerides; WC: waist circumference.

**Table 3:** Mean of the anthropometric variables according to the staging of NAFLD.

Variables	The staging of NAFLD by liver biopsy			Comparison of staging of NAFLD	*P* value
	Steatosis (*n* = 31)	Steatohepatitis without fibrosis (*n* = 17)	Steatohepatitis with fibrosis (*n* = 2)		
Weight (Kg)	120.4 ± 20.3	119.9 ± 19.7	148.5 ± 47.4	Steatosis × steatohepatitis without fibrosis	0.770
				Steatosis × steatohepatitis with fibrosis	0.209
				Steatohepatitis without fibrosis × steatohepatitis with fibrosis	0.225

WC (cm)	119.5 ± 11.1	123.8 ± 11.9	144.0 ± 18.3	Steatosis × steatohepatitis without fibrosis	0.719
				Steatosis × steatohepatitis with fibrosis	0.087
				Steatohepatitis without fibrosis × steatohepatitis with fibrosis	0.383

BMI (Kg/m^2^)	43.6 ± 3.4	40.5 ± 0.7	45.6 ± 2.2	Steatosis × steatohepatitis without fibrosis	0.189
				Steatosis × steatohepatitis with fibrosis	0.447
				Steatohepatitis without fibrosis × steatohepatitis with fibrosis	0.184

Mean and standard deviation; BMI: body mass index; WC: waist circumference.

**Table 4:** Mean serum concentrations of biochemical indicators of liver function and damage according to the staging of NAFLD.

Variables	The staging of NAFLD by liver biopsy			Comparison of staging of NAFLD	*P* value
	Steatosis (*n* = 31)	Steatohepatitis without fibrosis (*n* = 17)	Steatohepatitis with fibrosis (*n* = 2)		
AP (U/L)	75.1 ± 24.9	78.3 ± 20.2	73.1 ± 1.4	Steatosis × steatohepatitis without fibrosis	0.809
				Steatosis × steatohepatitis with fibrosis	0.892
				Steatohepatitis without fibrosis × steatohepatitis with fibrosis	0.767

AST (U/L)	22.9 ± 12.8	29.9 ± 26.7	30.2 ± 7.6	Steatosis × steatohepatitis without fibrosis	0.195
				Steatosis × steatohepatitis with fibrosis	0.547
				Steatohepatitis without fibrosis × steatohepatitis with fibrosis	0.484

ALT (U/L)	27.1 ± 15.5	28.2 ± 17.5	28.9 ± 10.3	Steatosis × steatohepatitis without fibrosis	0.216
				Steatosis × steatohepatitis with fibrosis	0.330
				Steatohepatitis without fibrosis × steatohepatitis with fibrosis	0.242

GGT (U/L)	29.4 ± 17.8	42.6 ± 27.7	62.4 ± 14.6	Steatosis × steatohepatitis without fibrosis	0.104
				Steatosis × steatohepatitis with fibrosis	**0.045**
				Steatohepatitis without fibrosis × steatohepatitis with fibrosis	0.566

Alb (g/dL)	4.5 ± 0.7	3.9 ± 0.3	3.3 ± 0.2	Steatosis × steatohepatitis without fibrosis	0.236
				Steatosis × steatohepatitis with fibrosis	**<0.001**
				Steatohepatitis without fibrosis × steatohepatitis with fibrosis	**0.003**

Mean and standard deviation; Alb: albumin; ALT: alanine aminotransferase; AP: alkaline phosphatase; AST: aspartate aminotransferase; GGT: gamma-glutamyl transpeptidase.

**Table 5:** Mean serum concentrations of MS and IR biochemical indicators according to the staging of NAFLD.

Variables	The staging of NAFLD by liver biopsy			Comparison of staging of NAFLD	*P* value
	Steatosis (*n* = 31)	Steatohepatitis without fibrosis (*n* = 17)	Steatohepatitis with fibrosis (*n* = 2)		
Glucose (mg/dL)	98.0 ± 12.4	91.5 ± 19.2	102.3 ± 12.0	Steatosis × steatohepatitis without fibrosis	0.395
				Steatosis × steatohepatitis with fibrosis	0.432
				Steatohepatitis without fibrosis × steatohepatitis with fibrosis	0.553

Insulin (mcU/mL)	16.7 ± 7.4	20.5 ± 13.2	24.0 ± 4.2	Steatosis × steatohepatitis without fibrosis	0.235
				Steatosis × steatohepatitis with fibrosis	**0.044**
				Steatohepatitis without fibrosis × steatohepatitis with fibrosis	0.874

HOMA-IR	3.7 ± 1.7	5.6 ± 6.1	6.4 ± 1.8	Steatosis × steatohepatitis without fibrosis	0.202
				Steatosis × steatohepatitis with fibrosis	0.076
				Steatohepatitis without fibrosis × steatohepatitis with fibrosis	0.239

TG (mg/dL)	118.1 ± 41.5	159.5 ± 43.3	181.5 ± 46.6	Steatosis × steatohepatitis without fibrosis	0.570
				Steatosis × steatohepatitis with fibrosis	**0.001**
				Steatohepatitis without fibrosis × steatohepatitis with fibrosis	0.437

HDL-c (mg/dL)	48.9 ± 12.3	42.9 ± 9.0	38.9 ± 7.2	Steatosis × steatohepatitis without fibrosis	0.307
				Steatosis × steatohepatitis with fibrosis	**0.038**
				Steatohepatitis without fibrosis × steatohepatitis with fibrosis	0.374

Mean and standard deviation; HDL-c: high-density lipoprotein cholesterol; HOMA-IR: homeostatic model assessment; TG: triglycerides.
